# Molecular Convergence Between Idiopathic Pulmonary Fibrosis and Its Comorbidities Reveals Interactions Between Pulmonary and Systemic Regulatory Programs

**DOI:** 10.3390/biology15131044

**Published:** 2026-06-30

**Authors:** Rafael Baltiérrez-Hoyos, Juan Manuel Velázquez-Enríquez, Jovito Cesar Santos-Álvarez, Dulce Natividad Jiménez-Gómez, Alma Aurora Ramírez-Hernández, Karina González-García, Cecilia Zertuche-Martínez, Itayetzi Reyes-Avendaño, Edilburga Reyes-Jiménez, Verónica Rocío Vásquez-Garzón

**Affiliations:** 1 Laboratorio de Fibrosis y Cáncer, Facultad de Medicina y Cirugía, Universidad Autónoma Benito Juárez de Oaxaca, Ex Hacienda de Aguilera S/N, Sur, San Felipe del Agua, Oaxaca 68020, Mexico; juanmanuelvela_enriquez@cecad-uabjo.mx (J.M.V.-E.); jovitocesarsa@cecad-uabjo.mx (J.C.S.-Á.); jimenezgomezdulcenatividad@gmail.com (D.N.J.-G.); aramih_09@outlook.com (A.A.R.-H.); k.igg@cecad-uabjo.mx (K.G.-G.); cecizertuche95@cecad-uabjo.mx (C.Z.-M.); itayetzi.reyes94@cecad-uabjo.mx (I.R.-A.); edilreyesjimnez@cecad-uabjo.mx (E.R.-J.); vrvasquezga@secihti.mx (V.R.V.-G.); 2 Directorate of Support for the Consolidation of the Scientific and Humanistic Community, Secretariat of Science, Humanities, Technology and Innovation—SECIHTI, Mexico City 03940, Mexico; 3 SECIHTI-Facultad de Medicina y Cirugía, Universidad Autónoma Benito Juárez de Oaxaca, Ex Hacienda de Aguilera S/N, Sur, San Felipe del Agua, Oaxaca 68020, Mexico; 4 Laboratorio de Biomedicina Traslacional y Sistemas, Centro de Investigación en Nutrición y Alimentación, Universidad del Istmo, Carretera Transísmica Juchitán, La Ventosa km. 14, La Ventosa, Oaxaca 70102, Mexico

**Keywords:** idiopathic pulmonary fibrosis, comorbidities, network analysis, lung cancer, cardiovascular disease, metabolic disorders, anxiety, type 2 diabetes, irritable bowel syndrome

## Abstract

Idiopathic pulmonary fibrosis (IPF) is a progressive interstitial lung disease characterized by high mortality and marked clinical heterogeneity. A defining yet insufficiently understood feature of IPF is its frequent coexistence with pulmonary and extrapulmonary comorbidities. However, the molecular basis that underlies this multisystem involvement remains unclear. We applied an integrative systems-level framework that combines lung-derived transcriptomic datasets with curated disease–gene associations across major IPF-associated comorbidities. Network organization and functional stratification analyses were used to characterize shared and disease-specific molecular modules, and independent transcriptomic datasets were analyzed to evaluate the consistency of these patterns. These analyses revealed structured molecular convergence between IPF and its comorbidities organized into two interacting biological programs: a lung-enriched fibrotic–mechanical program and a systemically distributed neuroimmune–metabolic program. These programs are interconnected through a shared regulatory interface that includes *IL6*, *AKT1*, *TGFB1* and *STAT3*. This organization was consistently observed across independent datasets. IPF emerges from the interaction between pulmonary and systemic regulatory programs rather than isolated lung-restricted mechanisms. This framework provides a basis for understanding disease heterogeneity and comorbidity burden, with potential implications for stratification and therapeutic targeting.

## Simple Summary

Idiopathic pulmonary fibrosis is a serious lung disease that causes progressive scarring of the lungs and is frequently accompanied by other conditions, such as heart disease, diabetes, digestive disorders, and anxiety. However, why these diseases commonly occur together remains unclear. In this study, we used a systems biology approach to examine how genes and molecular pathways associated with lung fibrosis interact with those linked to common comorbidities. By combining public genetic databases, lung transcriptomic data, and molecular interaction networks, we identified two major interconnected biological programs. One program is related mainly to lung tissue remodeling and mechanical stress, whereas the other program is associated with immune regulation, metabolism, and nervous system signaling across multiple organs. These programs are connected through shared regulatory molecules involved in inflammation and cell survival. Our findings suggest that idiopathic pulmonary fibrosis may be not only a lung-restricted disease but also a broader systemic disorder that involves coordinated molecular interactions throughout the body. This work may help improve the understanding of disease complexity, support the development of more integrated treatment strategies, and encourage future research into personalized approaches for patients with pulmonary fibrosis.

## 1. Introduction

Idiopathic pulmonary fibrosis (IPF) is a chronic, progressive interstitial lung disease that is characterized by irreversible scarring of the pulmonary parenchyma, which leads to respiratory failure and high mortality. Despite advances in antifibrotic therapies, disease progression remains highly variable, and treatment responses are heterogeneous [1,2]. This clinical variability suggests that IPF pathogenesis cannot be fully explained by lung-restricted fibrotic mechanisms alone, which highlights the need for broader frameworks that capture biological complexity beyond canonical pathways.

A defining yet underexplored feature of IPF is its frequent coexistence with multiple pulmonary and extrapulmonary comorbidities, including chronic obstructive pulmonary disease (COPD), lung cancer (LC), cardiovascular disease (CVD), metabolic disorders, and neuropsychiatric conditions (anxiety), which represent the most consistently reported manifestations in IPF cohorts [3,4]. These comorbidities substantially influence prognosis, quality of life, and therapeutic outcomes, yet they are commonly treated as independent clinical entities rather than as components of a shared biological landscape [5,6]. The molecular basis that underlies this comorbidity burden and its relationship to fibrosis progression remain poorly understood.

Previous molecular studies have provided important insights into IPF pathogenesis through the identification of dysregulated genes, pathways, and cell populations in fibrotic lung tissue [7,8,9,10]. However, most approaches have focused on single tissues, isolated disease comparisons, or direct gene overlap analyses. Although informative, such strategies may obscure nonrandom connectivity patterns by prioritizing differential expression magnitude over regulatory context and failing to distinguish ubiquitous inflammatory signals from disease-relevant convergence mechanisms. Consequently, the systemic dimensions of IPF and its associated comorbidities remain conceptually fragmented [11,12,13].

Network-based and systems biology approaches provide alternative frameworks for addressing this complexity by emphasizing molecular organization, interaction topology, and functional modularity rather than isolated molecular changes [14,15]. By integrating transcriptomic data across diseases and tissues, these approaches enable the identification of convergent signaling mediators and coordinated biological programs that may not be detectable through conventional analyses. Importantly, this perspective enables investigation of how lung-restricted pathological processes intersect with systemically distributed molecular networks, thus providing a structured means for examining multisystem involvement without presupposing causality [13]. The primary objective of this review is to apply an integrative systems biology approach to examine molecular convergence between IPF and its associated pulmonary and extrapulmonary comorbidities. By integrating publicly available lung tissue transcriptomic datasets derived from IPF cohorts, including RNA sequencing and independent microarray platforms, with curated disease–gene association resources [16,17,18,19,20,21,22], our objective is to delineate the shared and distinct molecular architectures that underlie IPF-associated disease states. Through this strategy, we seek to establish a coherent structure that clarifies how local fibrotic–mechanical processes interact with systemic neuroimmune–metabolic regulation, thus providing an interpretation of IPF heterogeneity and comorbidity burden.

## 2. Materials and Methods

### 2.1. Organization of Molecular Evidence That Links Ipf and Its Associated Comorbidities

In this study, an integrative synthesis of the available literature was combined with original computational analyses to explore molecular convergence between IPF and its associated pulmonary and extrapulmonary comorbidities. The analytical strategy was derived from publicly available RNA sequencing datasets and curated microarray-based signatures previously reported by our group [14,23,24,25]. To complement the narrative interpretation, these datasets were integrated to reconstruct protein–protein interaction networks, prioritize hub genes, identify functional modules, and contextualize transcriptomic patterns within a unified molecular framework. Molecular organization, network topology, and functional modularity, rather than isolated differential expression signals or direct cross-disease transcriptomic comparisons, were emphasized. This organizational strategy was intentionally designed to distinguish lung-restricted molecular alterations from systemically distributed molecular associations, without presupposing direct causal relationships between IPF and individual comorbidities. The resulting analytical structure provides a unified molecular space in which convergent patterns can be examined while preserving disease-specific contextual boundaries.

### 2.2. Identification of IPF- and Comorbidity-Associated Genes

Disease-associated genes for IPF and selected pulmonary (pulmonary arterial hypertension (PAH), COPD, and LC) and extrapulmonary comorbidities (coronary artery disease (CAD), type 2 diabetes (T2D), gastroesophageal reflux disease (GERD), anxiety, and irritable bowel syndrome (IBS)), which represent the most consistently reported manifestations in IPF cohorts, were retrieved from curated public databases, including GeneCards (https://www.genecards.org/) [16], the Comparative Toxicogenomics Database (CTD https://ctdbase.org/), [26], and DisGeNET (https://www.disgenet.org/) [17]. Database-specific relevance and evidence-based thresholds were applied to increase specificity and reduce noise. These disease–gene associations provide a structured representation of comorbidity-related molecular knowledge. For GeneCards, genes with a relevance score ≥ 10 were retained. In the CTD, only associations supported by direct evidence or curated references were included, thus excluding purely predictive annotations. In DisGeNET, disease–gene associations with a score ≥ 0.1 were selected. Only *Homo sapiens* entries were considered across all the databases.

### 2.3. Gene Standardization and Integration

Gene identifiers were standardized using UniProt mapping tools to ensure consistency across the databases. The retrieved gene lists were standardized using the UniProt Retrieve/ID mapping tool (https://www.uniprot.org/, accessed on 8 September 2025). Pseudogenes and nonmappable entries were excluded, and duplicate records were removed to generate nonredundant gene sets [18]. This harmonized gene universe served as the foundation for overlap analyses, network construction, and functional annotation.

### 2.4. Identification of Shared Molecular Signatures

Convergent molecular signatures between IPF and each comorbidity were identified through set intersection analyses. Venn diagrams were generated using Venny 2.1 to visualize overlaps (https://bioinfogp.cnb.csic.es/tools/venny/, accessed on 12 September 2025). Shared gene counts and Jaccard similarity indices were calculated as descriptive measures of molecular convergence and were subsequently used to define candidate gene sets for downstream network-based prioritization and functional characterization rather than as direct measures of pathogenic similarity. Because curated disease–gene association databases integrate evidence from multiple experimental and literature sources, substantial overlap may occur among complex multifactorial diseases and should therefore be interpreted within this context [15,24]. To generate the gene compartments used for downstream network analyses, pairwise overlap results were subsequently consolidated according to disease context. First, all genes shared between IPF and the selected pulmonary comorbidities were merged into a single nonredundant pulmonary-associated gene set, whereas genes shared between IPF and the selected extrapulmonary comorbidities were merged into a separate nonredundant systemic-associated gene set. These two consolidated gene collections were then compared through an additional overlap analysis, which yielded three final categories: pulmonary-enriched genes, extrapulmonary-associated genes, and shared genes (present in both collections). These three compartments constituted the basis for subsequent protein–protein interaction network reconstruction, hub prioritization, MCODE module detection, and functional enrichment analyses.

### 2.5. IPF Lung Transcriptomic Evidence and Cross-Platform Validation

Publicly available bulk transcriptomic datasets from IPF lung tissue and nondiseased controls were obtained from two sources: (i) RNA sequencing datasets from public repositories and (ii) curated microarray-based signatures from our previous integrative study [25,27,28]. Microarray-derived signatures were included as literature-based evidence without reprocessing, thereby enabling cross-platform contextualization while preserving original analytical frameworks.

### 2.6. Protein–Protein Interaction Network Analysis and Hub Prioritization

Protein–protein interaction (PPI) networks were constructed using the STRING application within Cytoscape (v3.10.2). Network topology metrics were used to identify highly connected nodes, and regulatory importance was emphasized over expression magnitude. Hub gene prioritization was performed using the cytoHubba plugin, and the maximal clique centrality (MCC) algorithm was applied to identify key integrative nodes within the network [20,29].

### 2.7. Network Module Detection, Functional Enrichment and Program-Level Interpretation

To further characterize network organization, module detection was performed using the Molecular Complex Detection (MCODE) plugin in Cytoscape. MCODE identifies densely interconnected regions on the basis of local connectivity density and vertex weighting, thereby enabling the detection of functional subnetworks. Default parameters were applied (degree cutoff = 2, node score cutoff = 0.2, K-core = 2, and max depth = 100). Modules were ranked by their MCODE scores, which reflect intracluster connectivity. The top-ranked modules were selected for downstream interpretation as intermediate organizational layers that link global network topology to biological function.

Functional enrichment analyses were performed using DAVID and ShinyGO to identify overrepresented gene ontology (GO) terms and KEGG pathways. GO enrichment included biological process (BP), molecular function (MF), and cellular component (CC) domains. KEGG pathway analysis was also performed. A significance threshold of *p* < 0.05 was applied. The results were interpreted at the level of coordinated biological programs, with particular emphasis on fibrotic remodeling, immune regulation, metabolic pathways, and system-level signaling [30,31,32].

### 2.8. Pulmonary and Systemic Classification Framework

To facilitate the biological interpretation of the identified molecular programs, genes were organized into pulmonary-enriched, extrapulmonary-associated, or shared categories according to their membership within the consolidated pulmonary-associated and extrapulmonary-associated gene collections generated during the analytical workflow. Specifically, all genes shared between IPF and the selected pulmonary comorbidities were merged into a nonredundant pulmonary-associated gene set, whereas all genes shared between IPF and the selected extrapulmonary comorbidities were merged into a separate nonredundant extrapulmonary-associated gene set. Comparison of these two consolidated collections yielded three analytical compartments: pulmonary-enriched genes (present only in the pulmonary-associated set), extrapulmonary-associated genes (present only in the extrapulmonary-associated set), and shared genes (present in both collections).

To facilitate reproducibility, the analytical workflow and operational definitions used for gene categorization are summarized in Supplementary Table S1. The table details the criteria by which genes were assigned to the pulmonary-enriched, extrapulmonary-associated, or shared compartments on the basis of consolidated overlap analyses and emphasizes that these categories represent context-dependent analytical groupings rather than intrinsic tissue specificity. Importantly, these categories were not intended to define tissue-exclusive expression or biological specificity. Rather, they provide an organizational framework for interpreting molecular programs in the context of IPF and its associated comorbidities. IPF lung transcriptomic evidence, proteomic detection in lung tissue, published information regarding tissue distribution, and network topology were subsequently used to biologically contextualize these compartments rather than to assign genes to them [14,28].

### 2.9. Statistical Analysis

No formal statistical inference was performed on the overlap magnitude, as curated disease–gene association databases integrate heterogeneous sources of evidence and do not constitute independent random gene sets suitable for permutation-based testing.

## 3. Results

### 3.1. Quantitative and Topological Integration of Curated Disease–Gene Associations Across Ipf and Comorbidities

**Figure 1:**
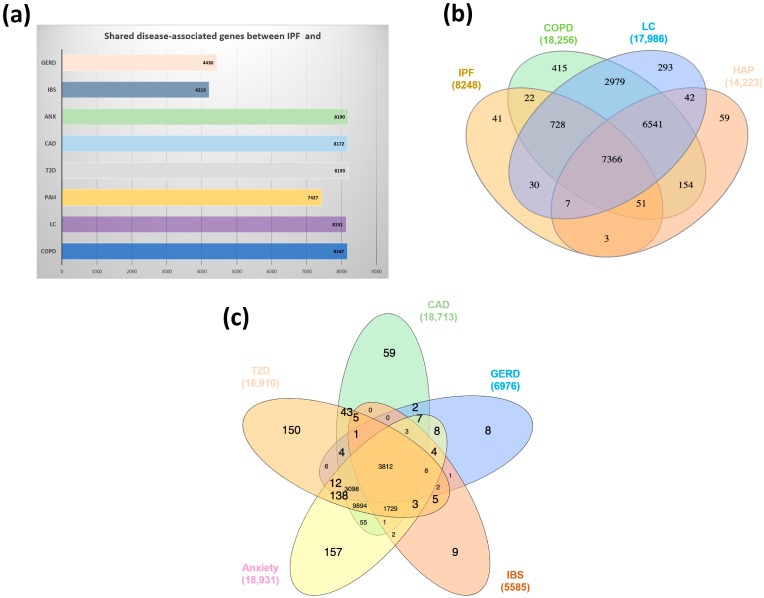
Gene overlap between pulmonary and extrapulmonary comorbidities associated with IPF. (**a**) Pairwise overlap analyses between idiopathic pulmonary fibrosis (IPF) and each pulmonary or extrapulmonary comorbidity, illustrating the extent of shared and disease-specific gene associations. (**b**) Venn diagram showing the consolidated overlap among pulmonary comorbidities. A total of 7366 genes were shared across pulmonary conditions. (**c**) Venn diagram showing the consolidated overlap among extrapulmonary comorbidities. A total of 3812 genes were shared across extrapulmonary conditions. Abbreviations: CAD, coronary artery disease; COPD, chronic obstructive pulmonary disease; GERD, gastroesophageal reflux disease; HAP, pulmonary arterial hypertension; IBS, irritable bowel syndrome; IPF, idiopathic pulmonary fibrosis; LC, lung cancer; and T2M, type 2 diabetes mellitus.

Comparative integration of curated disease–gene associations revealed extensive yet structurally asymmetric molecular overlap across IPF and its pulmonary and extrapulmonary comorbidities. After cross-database harmonization, the consolidated gene sets included 8248 genes for IPF, 18,256 genes for COPD, 17,986 genes for LC, 14,223 genes for PH, 18,910 genes for T2D, 18,713 genes for CAD, 18,931 genes for Anx, 5585 genes for IBS, and 6976 genes for GERD, which reflected differences in annotation depth and literature representation. Pairwise intersection analyses demonstrated high but nonuniform gene-level convergence among pulmonary diseases. The IPF–COPD comparison revealed 8167 shared genes, which corresponded to 99.0% of the IPF-associated genes and 44.7% of the COPD-associated genes. The IPF–LC intersection included 8131 shared genes (98.6% of the IPF genes and 45.2% of the LC genes), whereas IPF–PH included 7427 shared genes (90.0% of the IPF genes and 52.2% of the PH genes). Despite the large absolute overlaps, proportional similarity remained moderate. The Jaccard indices were 0.445 for IPF–COPD, 0.448 for IPF–LC, and 0.494 for IPF–PH, which indicated that less than half of the combined gene space was shared in each pairwise comparison. These values reveal marked structural asymmetry: although most IPF-associated genes overlap with genes associated with other pulmonary conditions, each comorbidity retains a substantial disease-specific molecular periphery (10,089 genes unique to COPD, 9855 genes unique to LC, and 6786 genes unique to PH) (Figure 1a). Cross-disease intersection analyses (Figure 1b) further revealed an expanded shared pulmonary gene core, which was consistent with a common lung-centered molecular architecture integrated within disease-specific peripheral regulatory landscapes, with 7366 genes shared.

A substantial proportion of the curated IPF-associated genes overlapped with those associated with several extrapulmonary comorbidities, which reflected an extensive shared molecular landscape derived from curated disease–gene association databases. This shared molecular space was subsequently refined through network topology, hub prioritization, module detection, and functional enrichment analyses to identify biologically relevant regulatory programs.

Integration with extrapulmonary conditions revealed a heterogeneous, quantitatively distinct convergence pattern (Figure 1a). The IPF–T2D comparison revealed 8193 shared genes, which corresponded to 99.3% of the IPF genes and 43.3% of the T2D genes, thus yielding a Jaccard index of 0.431. The IPF–CAD intersection included 8172 shared genes (99.1% of the IPF genes and 43.7% of the CAD genes), with a Jaccard index of 0.431. Similarly, 8190 genes (99.3% of the IPF genes and 43.2% of the Anx genes) were associated with IPF–anxiety disorders, with a Jaccard index of 0.430. In contrast, gastrointestinal comorbidities exhibited markedly reduced overlap. IPF–IBS shared 4213 genes (51.1% of the IPF genes and 75.4% of the IBS genes), which yielded a Jaccard index of 0.438, whereas IPF–GERD shared 4430 genes (53.7% of the IPF genes and 63.5% of the GERD genes), with a Jaccard index of 0.391. Unlike pulmonary diseases, several extrapulmonary multiple disease intersections (Figure 1c) further revealed an expanded shared pulmonary gene core comparison, which was consistent with a common lung-centered molecular backbone embedded within disease-specific peripheral architectures, with 3812 genes shared, thus reflecting greater structural divergence.

### 3.2. Cross-Disease Network Organization Reveals Structured Molecular Convergence

**Figure 2:**
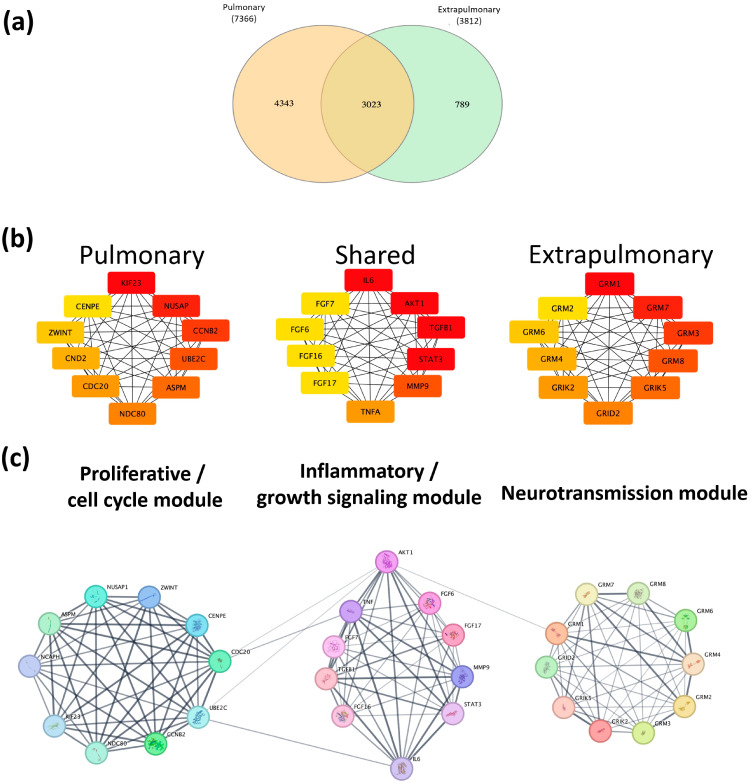
Network topology and identification of hub genes across pulmonary, shared, and extrapulmonary gene sets. (**a**) Venn diagram illustrating the distribution of genes between the consolidated pulmonary-associated and extrapulmonary-associated gene sets. Comparison of both collections identified 3023 shared genes, together with 4343 pulmonary-enriched genes and 789 extrapulmonary-associated genes. (**b**) Top 10 hub genes identified in each molecular compartment using the cytoHubba plugin based on the maximal clique centrality (MCC) algorithm. Pulmonary hubs were associated primarily with cell cycle regulation and mitotic processes (e.g., *KIF23*, *NUSAP1*, *CCNB2*, and *UBE2C*), shared hubs were enriched in inflammatory and fibrotic signaling (e.g., *IL6*, *AKT1*, *TGFB1*, and *STAT3*), whereas extrapulmonary hubs were dominated by glutamate receptor family members (e.g., *GRM* and *GRIK* family members). (**c**) Protein–protein interaction (PPI) subnetworks corresponding to the principal functional modules identified within each molecular compartment. These modules represent proliferative/cell cycle, inflammatory/growth signaling, and neurotransmission regulatory programs, respectively, supporting the existence of functionally distinct yet interconnected molecular domains. Red color represents a higher degree, and yellow color represents a lower degree.

To investigate the organizational properties that underlie shared molecular signatures, we constructed PPI networks using convergent gene sets derived from pulmonary and extrapulmonary comorbidities associated with IPF. Integration of disease-associated gene sets revealed 7366 pulmonary-related genes and 3812 extrapulmonary-related genes, with 3023 genes shared between both groups, which indicated a central layer of molecular convergence. Additionally, 4343 genes were uniquely associated with pulmonary comorbidities, whereas 789 genes were exclusive to extrapulmonary conditions (Figure 2a). Detailed lists of these genes are provided in Supplementary Table S2.

These three gene subsets (pulmonary-specific, shared, and extrapulmonary-specific) were imported into Cytoscape (v3.10.2) using the STRING database to construct protein–protein interaction (PPI) networks and evaluate their topological organization. Network analysis revealed nonrandom connectivity patterns characterized by dense clustering and the emergence of highly interconnected regions rather than a uniform distribution across nodes (Figure S1). This organization supports the presence of structured cross-disease interaction patterns, which are defined here as coordinated interaction patterns that involve multiple interconnected nodes that collectively shape network behavior, rather than isolated gene-level effects. Within these structures, highly connected nodes occupied central topological positions, which suggests their role as integrative functional elements within the molecular system. This global architecture established a structural framework for subsequent module detection and functional stratification.

In the pulmonary network, the top-ranked hub genes included *KIF23*, *NUSAP1*, *CCNB2*, *UBE2C*, *ASPM*, *NDC80*, *CDC20*, *CCND2*, *ZWINT*, and *CENPE*, which reflected strong enrichment in the mitotic machinery and proliferative signaling. In the shared network, the hub genes were *IL6*, *AKT1*, *TGFB1*, *STAT3*, *MMP9*, *TNF*, *FGF17*, *FGF16*, *FGF6*, and *FGF7*, which represented a convergence of inflammatory signaling, growth factor pathways, and extracellular matrix (ECM) remodeling, consistent with an interface that links fibrosis and systemic responses. In contrast, the extrapulmonary network was dominated by hub genes that belong to the glutamate receptor family, including *GRM1*, *GRM7*, *GRM3*, *GRM8*, *GRIK5*, *GRID2*, *GRIK2*, *GRM4*, *GRM6*, and *GRM2*, thus indicating strong enrichment in neurotransmitter signaling pathways (Figure 2b). The complete MCC rankings for the three networks are provided in Supplementary Table S3.

In addition to identifying pulmonary-specific and extrapulmonary-specific network architectures, we performed a protein–protein interaction (PPI) analysis of the hub genes and revealed a central layer of shared molecular connectivity, which was defined by genes common to both domains. Rather than representing simple overlap, this shared component exhibited a distinct topological organization enriched in highly interconnected central signaling pathways, including key mediators such as IL6, AKT1, TGFB1, and STAT3. These nodes occupied central positions that were linked to otherwise functionally distinct network regions (Figure 2c).

This intermediate layer is therefore conceptualized as a shared regulatory interface that represents a structured molecular space that integrates pulmonary and systemic signaling. Within this interface, inflammatory, growth factor, and stress-response pathways converge, thereby enabling coordinated communication between lung-restricted and systemically distributed processes. This organization supports a hierarchical network model in which disease-associated molecular architecture is not binary but instead structured across interconnected layers, thus providing a basis for functional stratification into distinct but interacting biological programs.

### 3.3. Functional Stratification Reveals Fibrotic–Mechanical and Neuroimmune–Metabolic Programs Interconnected Through a Shared Regulatory Interface

**Figure 3:**
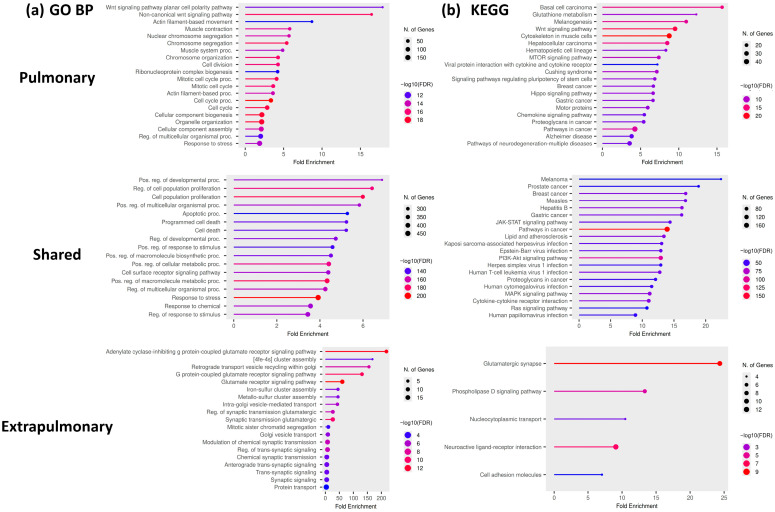
Functional organization of convergent gene networks into interconnected regulatory programs (**a**) Gene Ontology Biological Process (GO BP) enrichment analysis of the pulmonary-enriched, shared, and extrapulmonary-associated gene sets. Pulmonary-associated genes were enriched primarily in cell cycle regulation, cytoskeletal organization, and tissue remodeling processes, whereas shared and extrapulmonary-associated gene sets were enriched in inflammatory, neuroimmune, metabolic, and synaptic signaling processes. (**b**) KEGG pathway enrichment analysis of the corresponding gene sets. Pulmonary-associated genes were predominantly associated with proliferative and cancer-related signaling pathways, shared genes with inflammatory and immune signaling pathways, and extrapulmonary-associated genes with neurotransmission and glutamatergic signaling pathways. Together, these enrichment analyses support the existence of interconnected pulmonary fibrotic–mechanical and systemic neuroimmune–metabolic regulatory programs rather than independent biological processes. Abbreviation: proc., process.

Functional enrichment analysis of network-organized gene sets revealed the emergence of two major biological programs, which were interconnected through a central interface rather than operating as independent systems. The first program, which was derived primarily from the pulmonary-specific network, was characterized by enrichment of processes related to ECM organization, cytoskeletal dynamics, cell cycle progression, and mechanotransduction. These features define a fibrotic–mechanical program, which is consistent with lung-restricted tissue remodeling and mechanical stress responses that are characteristic of IPF pathology. In contrast, the second program, which was associated predominantly with the extrapulmonary network, was enriched for immune regulation, neurotransmitter signaling, metabolic pathways, and stress-response processes, thus defining a neuroimmune–metabolic program with broad systemic distribution. Critically, these two programs were functionally linked through a shared regulatory interface composed of genes common to both pulmonary and extrapulmonary networks. Functional enrichment of this shared layer revealed a convergence of cytokine signaling, growth factor pathways, and integrative stress-response mechanisms (Figure 3a,b). Detailed gene lists and functional enrichment data are provided in Supplementary Tables S4–S7 and Figure S2.

Key nodes within this interface, including IL6, AKT1, TGFB1, and STAT3, suggest that this layer operates as a bidirectional communication axis that coordinates local fibrotic remodeling with systemic molecular inputs. Together, these findings support a three-layered organizational model that comprises a lung-restricted fibrotic–mechanical program, a systemically distributed neuroimmune–metabolic program, and a shared regulatory interface that functionally integrates these programs. This integrative organization is summarized schematically, illustrating the reciprocal interaction among local fibrotic remodeling, systemic regulatory processes, and their common signaling interface. This model provides a mechanistic basis for understanding IPF not as an isolated pulmonary disorder but as a disease embedded within a broader multisystem regulatory network.

### 3.4. Transcriptomic Contextualization Supports Regulatory Prioritization Beyond Expression Magnitude

**Figure 4:**
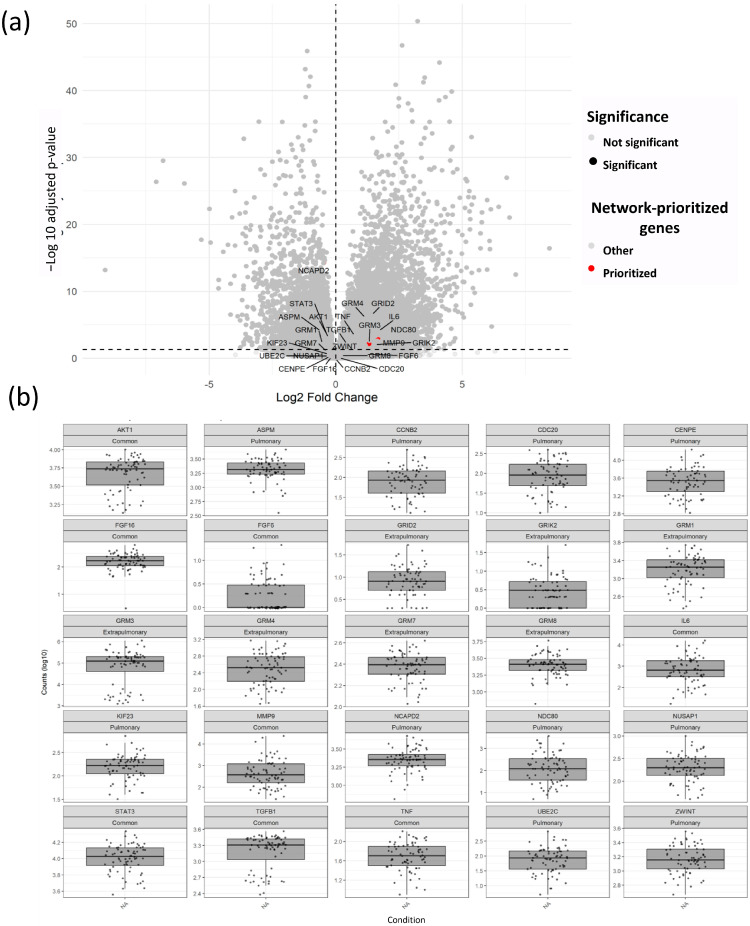
Transcriptomic landscape of IPF lung tissue with prioritized genes highlighted. (**a**) Volcano plot showing differential gene expression between IPF and control lung tissues. Genes prioritized through the network-based analysis are highlighted and distributed across a broad range of fold changes and statistical significance, illustrating that network centrality and disease convergence are not restricted to the most highly differentially expressed transcripts. (**b**) Boxplots showing normalized expression levels of representative network-prioritized genes across control and IPF lung tissue samples. The selected genes exhibit heterogeneous expression patterns, including consistent, moderate, and variable changes, supporting the concept that regulatory importance reflects coordinated network organization rather than transcriptional magnitude alone.

To contextualize prioritized genes within the transcriptional landscape of IPF, differential expression patterns were examined in lung tissue datasets. The global distribution of gene expression changes is shown in a volcano plot, where selected genes are highlighted within the broader transcriptomic context (Figure 4a). Notably, the prioritized genes were not exclusively among the most strongly differentially expressed features. Instead, they were distributed across a range of expression values, including both significantly and moderately altered transcripts. This pattern reflects the selection strategy used in this study, which prioritized genes on the basis of network centrality and cross-disease convergence rather than differential expression magnitude alone. Importantly, the purpose of this analysis was not to provide independent functional validation of network-prioritized genes but rather to illustrate the complementary relationship between transcriptomic differential expression and network topology. To further examine gene-level behavior, the expression profiles of selected genes were evaluated across conditions (Figure 4b). These analyses revealed heterogeneous expression patterns, with some genes displaying consistent upregulation, whereas others exhibited moderate or variable expression levels, thus suggesting context-dependent activity. This variability indicates that the molecular programs identified in this study are driven not only by large transcriptional shifts but also by coordinated interactions embedded within the network structure.

Importantly, several of the prioritized genes occupy central positions within PPI networks and are functionally linked to key signaling pathways, despite not exhibiting the greatest changes in expression. This observation supports the notion that regulatory influence within complex biological systems is not proportional to transcriptional amplitude but instead reflects the integration of signaling dynamics and network connectivity. Collectively, these findings reinforce the interpretation that the molecular architecture that underlies IPF and its associated comorbidities is defined by coordinated regulatory organization rather than by isolated high-magnitude transcriptional changes. These findings further illustrate that transcriptomic magnitude and network centrality provide complementary layers of biological information, thus reinforcing the value of integrating expression data with network organization to identify biologically meaningful regulatory programs.

## 4. Discussion

IPF is increasingly recognized as a complex disease that is frequently accompanied by cardiovascular, metabolic, gastrointestinal, and neuropsychiatric comorbidities, which suggests that fibrotic lung remodeling occurs within a broader systemic regulatory context rather than as an isolated organ-specific process [4,5,33,34]. Clinical cohort studies have consistently shown that these comorbidities influence disease progression, quality of life, and survival; however, their mechanistic integration into IPF pathobiology remains incomplete [34].

Most prior molecular investigations have focused on single tissues, isolated disease comparisons, or direct gene overlap analyses. Although these approaches have provided valuable insights into disease-associated pathways, they often obscure structured network organization by prioritizing differential expression magnitude and failing to distinguish ubiquitous inflammatory signals from disease-relevant convergence mechanisms [15,24]. As a result, the systemic dimensions of IPF and its associated comorbidities remain conceptually fragmented. In this context, the present findings support a shift from gene-centric and tissue-restricted interpretations toward an integrated signaling-based view in which disease phenotypes emerge from coordinated interactions. This perspective aligns with emerging clinical evidence that demonstrates that progressive pulmonary phenotypes and their severity are strongly modulated by the cumulative effect of chronic, systemic, and historical inflammatory insults rather than by isolated organ-restricted mechanisms. This perspective emphasizes coordinated signaling interactions and pathway integration rather than isolated molecular alterations. Network-based approaches provide an alternative perspective by emphasizing molecular organization, interaction topology, and functional modularity rather than isolated molecular changes [14,35]. Disease network analyses have demonstrated that complex disorders frequently share profibrotic and inflammatory signaling pathways, even when direct gene overlap is modest [24]. Integrating transcriptomic signals across pulmonary and extrapulmonary disease states enables the identification of convergent molecular architectures that are not apparent through conventional reductionist strategies.

Importantly, the extensive gene overlap observed between IPF and several associated comorbidities should be interpreted in the context of the curated databases used in this study. Resources such as GeneCards, CTD, and DisGeNET integrate evidence from multiple experimental and literature-derived sources and therefore capture broad molecular associations across complex multifactorial diseases [15,24]. Consequently, overlap magnitude does not necessarily imply direct pathogenic equivalence but rather defines a shared molecular space that requires subsequent network-based prioritization and functional interpretation. In future studies, confidence-weighted evidence, database-specific filtering strategies, or experimentally validated disease–gene associations can be incorporated to refine these molecular relationships and improve disease-specific resolution.

Within this framework, we delineate a lung-enriched fibrotic–mechanical program characterized by genes involved in cytoskeletal organization, ECM remodeling, and proliferative signaling. These processes are well aligned with current concepts of fibrosis as a mechanically reinforced pathological state, in which increased matrix stiffness amplifies profibrotic signaling through mechanotransduction pathways, thereby creating self-sustaining feedback loops within lung tissue [8,36]. In addition, excessive collagen deposition progressively alters the biomechanical properties of the distal lung and exposes epithelial cells and fibroblasts to abnormal mechanical forces that reshape cellular behavior and tissue organization [8]. Such mechanically driven programs provide a plausible explanation for the persistence and progression of fibrosis despite the removal of initiating insults.

Mechanical stress generated by matrix stiffening is sensed through integrin-mediated adhesions and cytoskeletal tension systems. Integrins such as αvβ6 participate in force transmission and the activation of latent TGF-β, thereby coupling ECM mechanics to profibrotic signaling pathways [37]. Activation of FAK and downstream mechanotransduction pathways promotes fibroblast survival, migration, and myofibroblast differentiation, thereby ultimately sustaining excessive ECM production. In parallel, matrix rigidity amplifies mechanosensitive transcriptional programs mediated by YAP and TAZ, thus reinforcing profibrotic gene expression, cellular contractility, and fibroblast persistence [38]. Within this mechanically altered niche, fibroblasts acquire apoptosis resistance and increased contractile capacity, which promotes the persistence of activated myofibroblast populations that drive irreversible tissue remodeling. Importantly, fibroblast activation occurs through close interactions with injured alveolar epithelial cells. Alveolar type II epithelial cells exposed to persistent mechanical stress exhibit impaired regenerative capacity, altered epithelial homeostasis, and increased secretion of profibrotic mediators, including TGF-β, IL6, and CTGF. This dysfunctional epithelial state may establish maladaptive epithelial–mesenchymal feedback loops that perpetuate fibrosis progression and defective tissue repair [39]. Emerging evidence further suggests that inflammatory and fibrotic pathways become functionally interconnected within the mechanically altered lung microenvironment. Chronic cytokine signaling, including IL6/STAT3 and PI3K/AKT activation, contributes to fibroblast survival, immune amplification, and metabolic adaptation within the fibrotic niche [7]. Profibrotic macrophage populations may further reinforce ECM remodeling and chronic inflammatory activation, thus contributing to the persistence of maladaptive repair responses. Together, these findings support a model in which IPF progression is sustained by a mechanobiological reinforcement system that involves epithelial dysfunction, fibroblast persistence, inflammatory amplification, and ECM remodeling. This self-sustaining pathological circuit ultimately promotes irreversible distortion of pulmonary architecture, progressive loss of lung compliance, and impaired gas exchange, thus linking molecular network organization with tissue-level physiological dysfunction.

In parallel, we identified a systemically distributed neuroimmune–metabolic program enriched for pathways related to neurotransmitter signaling, immune regulation, and metabolic control. Neuroimmune circuits, including those involved in vagal and stress-responsive pathways, are being increasingly recognized as key regulators of systemic inflammatory tone and immune homeostasis [40]. Consistent with this observation, the extrapulmonary regulatory network was notably enriched in glutamate receptor family members, including multiple metabotropic (*GRM1*, *GRM2*, *GRM3*, *GRM4*, *GRM6*, *GRM7*, and *GRM8*) and ionotropic (*GRIK2*, *GRIK5*, and *GRID2*) glutamate receptors. Although these molecules are not currently recognized as canonical drivers of IPF pathogenesis, accumulating evidence indicates that glutamatergic signaling is involved in neuroimmune communication, immune cell regulation, and metabolic homeostasis beyond the central nervous system [41]. Therefore, their emergence as highly connected hub genes in our network should be regarded as a hypothesis-generating observation that suggests that glutamatergic pathways may contribute to the systemic regulatory architecture that links IPF with its extrapulmonary comorbidities rather than representing direct evidence of a causal role in pulmonary fibrosis. Similarly, immunometabolic signaling networks coordinate energy balance and inflammatory responses across tissues, thereby contributing to chronic disease susceptibility and multisystem involvement [42]. The broad tissue distribution reported for proteins within this program in reference atlases indicates that these genes are not lung-restricted. Importantly, their classification as extrapulmonary reflects systemic regulatory associations on the basis of expression breadth and network context rather than direct transcriptomic or functional validation in extrapulmonary tissues. Importantly, these fibrotic–mechanical and neuroimmune–metabolic programs are not independent but intersect at shared signaling mediators involved in inflammation, survival signaling and mechanotransduction. Rather than implying a unidirectional causal relationship, this intersection supports a model of reciprocal modulation in which lung-restricted pathology both influences and is influenced by systemically distributed networks [23,35]. This perspective aligns with emerging views of IPF as a heterogeneous disease shaped by interactions between local mechanobiological remodeling and systemic inflammatory signaling [43].

On the basis of the integrative evidence generated in this study, we propose that IPF can be conceptualized as a “pathological systems attractor”, which is defined here as a relatively stable disease state sustained by the coordinated interaction of interconnected molecular networks and regulatory programs rather than by the dysregulation of a single pathway. This proposal builds upon attractor theory in complex biological systems, which was originally introduced in the context of gene regulatory networks and self-organizing biological systems by Kauffman [44] and subsequently extended to cellular state transitions and disease biology by Huang and colleagues [45,46]. More recently, attractor theory has been applied to complex human disorders, including major depressive disorder and Alzheimer’s disease, where pathological phenotypes have been conceptualized as stable and self-reinforcing network configurations maintained by interacting biological processes [47,48].

**Figure 5:**
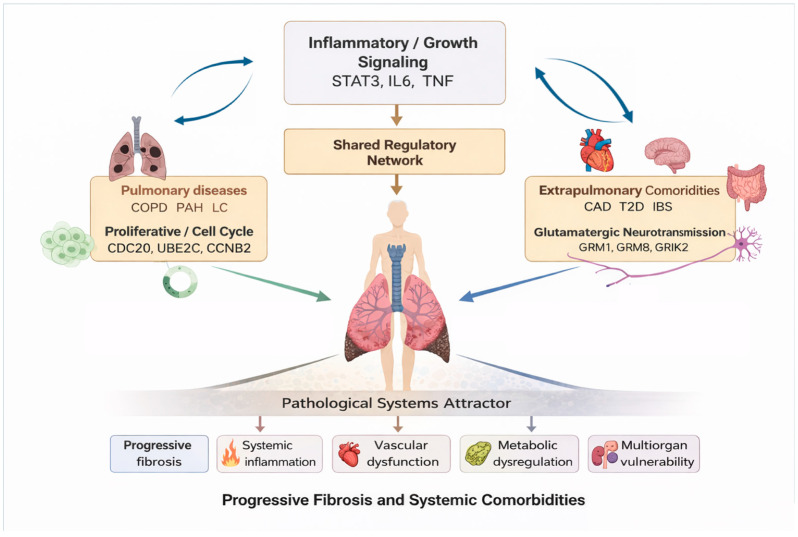
Dual-module system architecture that links pulmonary fibrosis and systemic comorbidities in IPF. Schematic representation of the proposed framework in which molecular convergence between IPF and its associated comorbidities is organized into two interacting biological programs. The pulmonary fibrotic–mechanical program involves ECM remodeling, cytoskeletal dynamics and proliferative signaling, thus reflecting lung-restricted processes. The systemically distributed neuroimmune–metabolic program includes immune regulation, neurotransmitter signaling and metabolic pathways. These programs are interconnected through a shared regulatory interface enriched in key signaling mediators, including IL6, AKT1, TGFB1 and STAT3. Bidirectional interactions between modules suggest coordinated regulation between local tissue remodeling and systemic molecular networks. This organization provides a systems-level basis for understanding disease heterogeneity and multisystem involvement in IPF.

Within this conceptual framework, our findings suggest that pulmonary fibrotic–mechanical and systemic neuroimmune–metabolic programs are interconnected through a shared regulatory interface that may contribute to the persistence of disease-associated network states (Figure 5). Rather than representing isolated pathogenic mechanisms, these coordinated programs may collectively stabilize the molecular organization that underlies chronic disease progression and multisystem involvement. Accordingly, the proposed “pathological systems attractor” should be regarded as an integrative and hypothesis-generating conceptual model that provides a unified perspective for understanding IPF as a multisystem disorder and offers a theoretical framework for the future development of network-informed therapeutic strategies.

From a clinical perspective, this integrative framework provides a coherent explanation for the high incidence and strong effects of comorbidities in IPF patients, thus suggesting that these conditions may arise from shared molecular mechanisms rather than independent co-occurring diseases. This interpretation has potential implications for patient stratification and suggests that distinct clinical trajectories may arise from differences in the relative contribution of pulmonary and systemic regulatory programs. In addition, it supports the exploration of therapeutic strategies that target shared signaling pathways that operate across tissues rather than focusing exclusively on lung-restricted mechanisms.

In this study, an integrative analytical approach was applied to identify convergent molecular architectures across IPF and its associated comorbidities. Accordingly, the analyses did not aim to establish direct causal relationships or temporal hierarchies between pulmonary and extrapulmonary processes. Instead, the identified programs reflect coordinated patterns of molecular organization derived from transcriptomic integration and network topology. Several limitations should be considered. First, the transcriptomic analyses were restricted to lung tissue, thus limiting direct assessment of molecular activity in extrapulmonary organs; consequently, independent transcriptomic or proteomic validation in extrapulmonary tissues remains a high priority for future studies to definitively confirm this systemic hypothesis. Second, disease–gene associations were derived from curated databases, which may have introduced biases related to data availability and curation criteria. Third, the integration of heterogeneous public datasets introduced variability in terms of platform technologies, cohort composition, and clinical annotation. Additionally, the overlap analyses were intended as descriptive representations of shared molecular associations and were not subjected to permutation- or enrichment-based statistical testing. Consequently, the biological conclusions of this work rely primarily on network topology, module organization, hub prioritization, and functional enrichment rather than on overlap magnitude. Finally, systematic protein-level validation was not systematically performed, and the available datasets reflect steady-state conditions rather than dynamic responses.

## 5. Conclusions

IPF may be better understood as a multisystem network disease that arises from the interaction between lung-restricted fibrotic processes and systemically distributed regulatory programs. Within this framework, IPF-associated comorbidities are not merely co-occurring conditions but represent manifestations of shared molecular organization embedded in a broader regulatory system. The conceptual model presented in Figure 5 integrates these findings into a unified systems-level framework and highlights the bidirectional relationship between pulmonary remodeling and systemic regulatory networks. This perspective provides a conceptual basis for interpreting disease heterogeneity and suggests that targeting shared regulatory pathways may offer new opportunities for therapeutic intervention beyond organ-restricted approaches.

## Data Availability

No new data were created or analyzed in this study.

## Supplementary Materials

The following supporting information can be downloaded at https://www.mdpi.com/article/10.3390/biology15131044/s1: Figure S1: Global protein–protein interaction network of integrated gene sets; Figure S2: Functional enrichment analysis of integrated gene sets; Table S1: Operational criteria for gene classification used in downstream network analyses. Table S2: Gene sets associated with pulmonary, extrapulmonary, and shared comorbidities; Table S3: STRING network MCC top10; Table S4: Gene ontology enrichment analysis of pulmonary-associated genes; Table S5: Gene ontology enrichment analysis of shared gene sets; Table S6: Gene ontology enrichment analysis of extrapulmonary-associated genes; and Table S7: Top MCODE modules identified within the PPI network.

## Abbreviations

The following abbreviations are used in this manuscript:

AKT1	AKT Serine/Threonine Kinase 1
ASPM	Abnormal Spindle Microtubule Assembly
BP	Biological process
CAD	Coronary artery disease
CC	Cellular component
CCNB2	Cyclin B2
CCND2	Cyclin D2
CDC20	Cell Division Cycle 20
CENPE	Centromere Protein E
COPD	Chronic obstructive pulmonary disease
CTD	Comparative Toxicogenomics Database
CVD	Cardiovascular disease
FGF16	Fibroblast Growth Factor 16
FGF17	Fibroblast Growth Factor 17
FGF6	Fibroblast Growth Factor 6
FGF7	Fibroblast Growth Factor 7
GERD	Gastroesophageal reflux disease
GO	Gene Ontology
GRID2	Glutamate Ionotropic Receptor Delta Type Subunit 2
GRIK2	Glutamate Ionotropic Receptor Kainate Type Subunit 2
GRIK5	Glutamate Ionotropic Receptor Kainate Type Subunit 5
GRM1	Glutamate Metabotropic Receptor 1
GRM2	Glutamate Metabotropic Receptor 2
GRM3	Glutamate Metabotropic Receptor 3
GRM4	Glutamate Metabotropic Receptor 4
GRM6	Glutamate Metabotropic Receptor 6
GRM7	Glutamate Metabotropic Receptor 7
GRM8	Glutamate Metabotropic Receptor 8
IBS	Irritable bowel syndrome
IL6	Interleukin 6
IPF	Idiopathic pulmonary fibrosis
KIF23	Kinesin Family Member 23
LC	Lung cancer
MCC	Maximal clique centrality
MCODE	Molecular Complex Detection
MF	Molecular function
MMP9	Matrix Metallopeptidase 9
NDC80	NDC80 Kinetochore Complex Component
NUSAP1	Nucleolar and Spindle Associated Protein 1
PAH	Pulmonary arterial hypertension
PPI	Protein–protein interaction
STAT3	Signal Transducer and Activator of Transcription 3
T2D	Type 2 diabetes
TGFB1	Transforming Growth Factor Beta 1
TNF	Tumor Necrosis Factor
UBE2C	Ubiquitin Conjugating Enzyme E2 C
ZWINT	ZW10 Interacting Kinetochore Protein
