# Supplementary material for: Molecular Convergence Between Idiopathic Pulmonary Fibrosis and Its Comorbidities Reveals Interactions Between Pulmonary and Systemic Regulatory Programs
Source: Biology (Basel). 2026 Jun 30;15(13):1044. doi: 10.3390/biology15131044 (PMC13359400; doi:10.3390/biology15131044)

Supplementary Figures

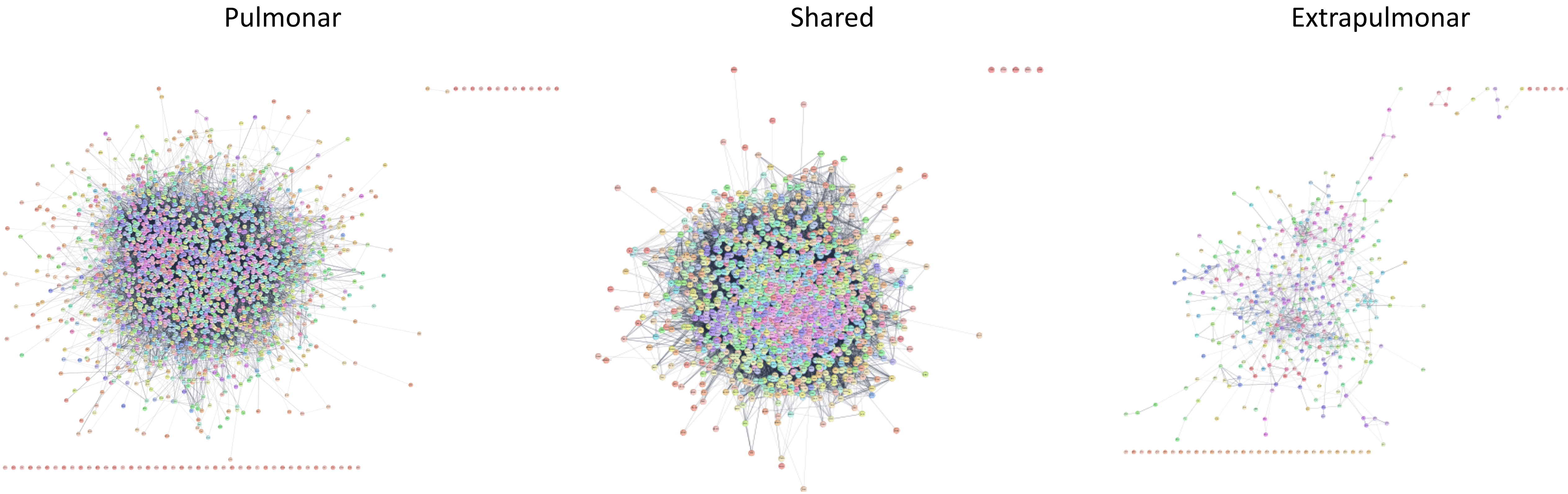

Figure S1. Global protein–protein interaction network of integrated gene sets
Full PPI network generated from pulmonary, common, and extrapulmonary gene sets, illustrating overall network topology and connectivity beyond highlighted hub genes.

Figure S2. Functional enrichment analysis of integrated gene sets

Bubble plots representing Gene Ontology (GO) biological processes (BP), celular comparment (CC), and molecullar function (MF) and KEGG pathways enriched in pulmonary, shared, and extrapulmonary gene groups.

PULMONAR

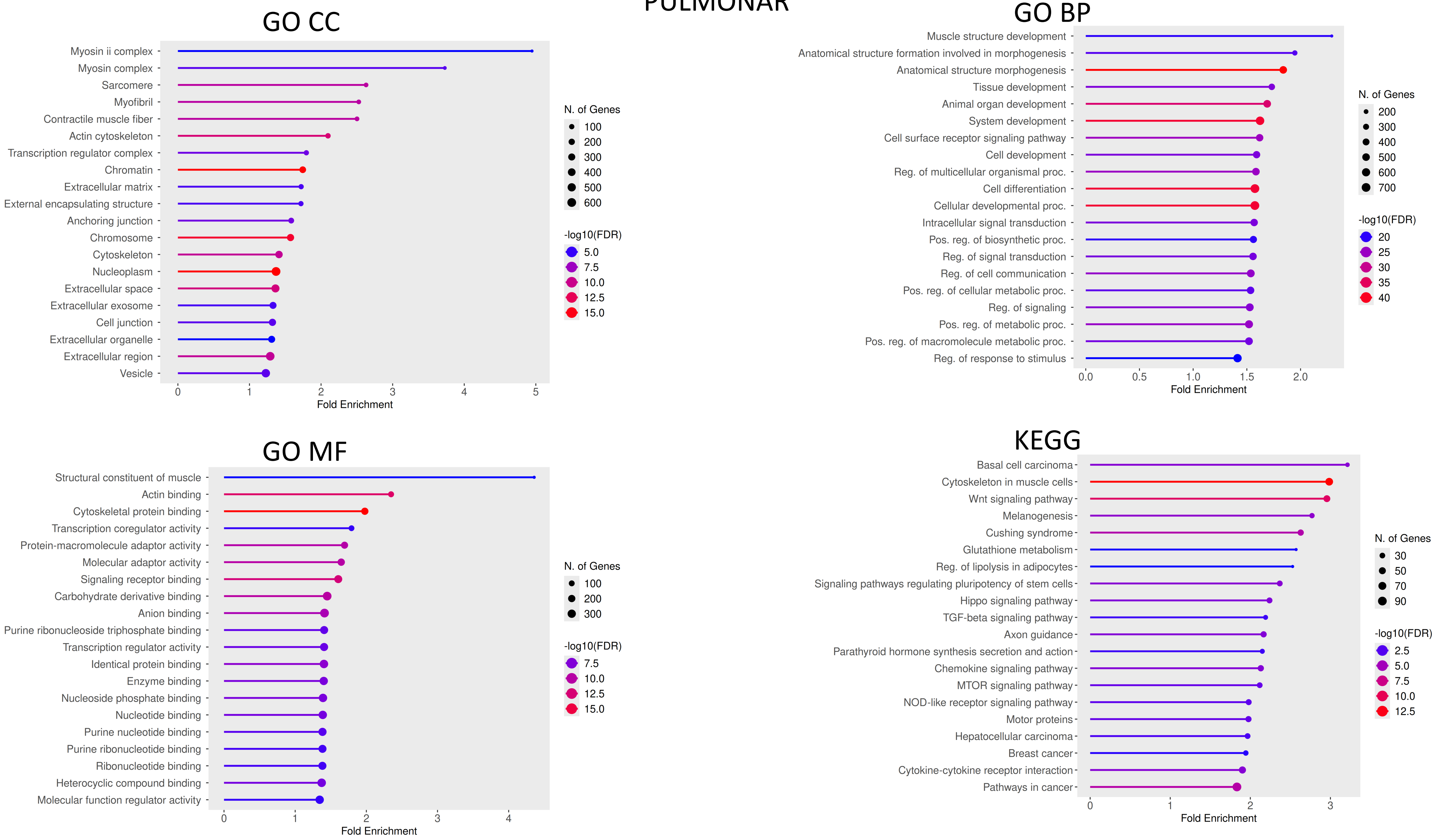

GO CC
Myosin ii complex
Myosin complex
Sarcomere
Myofibril
Contractile muscle fiber
Actin cytoskeleton
Transcription regulator complex
Chromatin
Extracellular matrix
External encapsulating structure
Anchoring junction
Chromosome
Cytoskeleton
Nucleoplasm
Extracellular space
Extracellular exosome
Cell junction
Extracellular organelle
Extracellular region
Vesicle
0 1 2 3 4 5
Fold Enrichment
N. of Genes
100
200
300
400
500
600
-log10(FDR)
5.0
7.5
10.0
12.5
15.0
GO BP
Muscle structure development
Anatomical structure formation involved in morphogenesis
Anatomical structure morphogenesis
Tissue development
Animal organ development
System development
Cell surface receptor signaling pathway
Cell development
Reg. of multicellular organismal proc.
Cell differentiation
Cellular developmental proc.
Intracellular signal transduction
Pos. reg. of biosynthetic proc.
Reg. of signal transduction
Reg. of cell communication
Pos. reg. of cellular metabolic proc.
Reg. of signaling
Pos. reg. of metabolic proc.
Pos. reg. of macromolecule metabolic proc.
Reg. of response to stimulus
0.0 0.5 1.0 1.5 2.0
Fold Enrichment
N. of Genes
200
300
400
500
600
700
-log10(FDR)
20
25
30
35
40
GO MF
Structural constituent of muscle
Actin binding
Cytoskeletal protein binding
Transcription coregulator activity
Protein-macromolecule adaptor activity
Molecular adaptor activity
Signaling receptor binding
Carbohydrate derivative binding
Anion binding
Purine ribonucleoside triphosphate binding
Transcription regulator activity
Identical protein binding
Enzyme binding
Nucleoside phosphate binding
Nucleotide binding
Purine nucleotide binding
Purine ribonucleotide binding
Ribonucleotide binding
Heterocyclic compound binding
Molecular function regulator activity
0 1 2 3 4
Fold Enrichment
N. of Genes
100
200
300
-log10(FDR)
7.5
10.0
12.5
15.0
KEGG
Basal cell carcinoma
Cytoskeleton in muscle cells
Wnt signaling pathway
Melanogenesis
Cushing syndrome
Glutathione metabolism
Reg. of lipolysis in adipocytes
Signaling pathways regulating pluripotency of stem cells
Hippo signaling pathway
TGF-beta signaling pathway
Axon guidance
Parathyroid hormone synthesis secretion and action
Chemokine signaling pathway
MTOR signaling pathway
NOD-like receptor signaling pathway
Motor proteins
Hepatocellular carcinoma
Breast cancer
Cytokine-cytokine receptor interaction
Pathways in cancer
0 1 2 3
Fold Enrichment
N. of Genes
30
50
70
90
-log10(FDR)
2.5
5.0
7.5
10.0
12.5

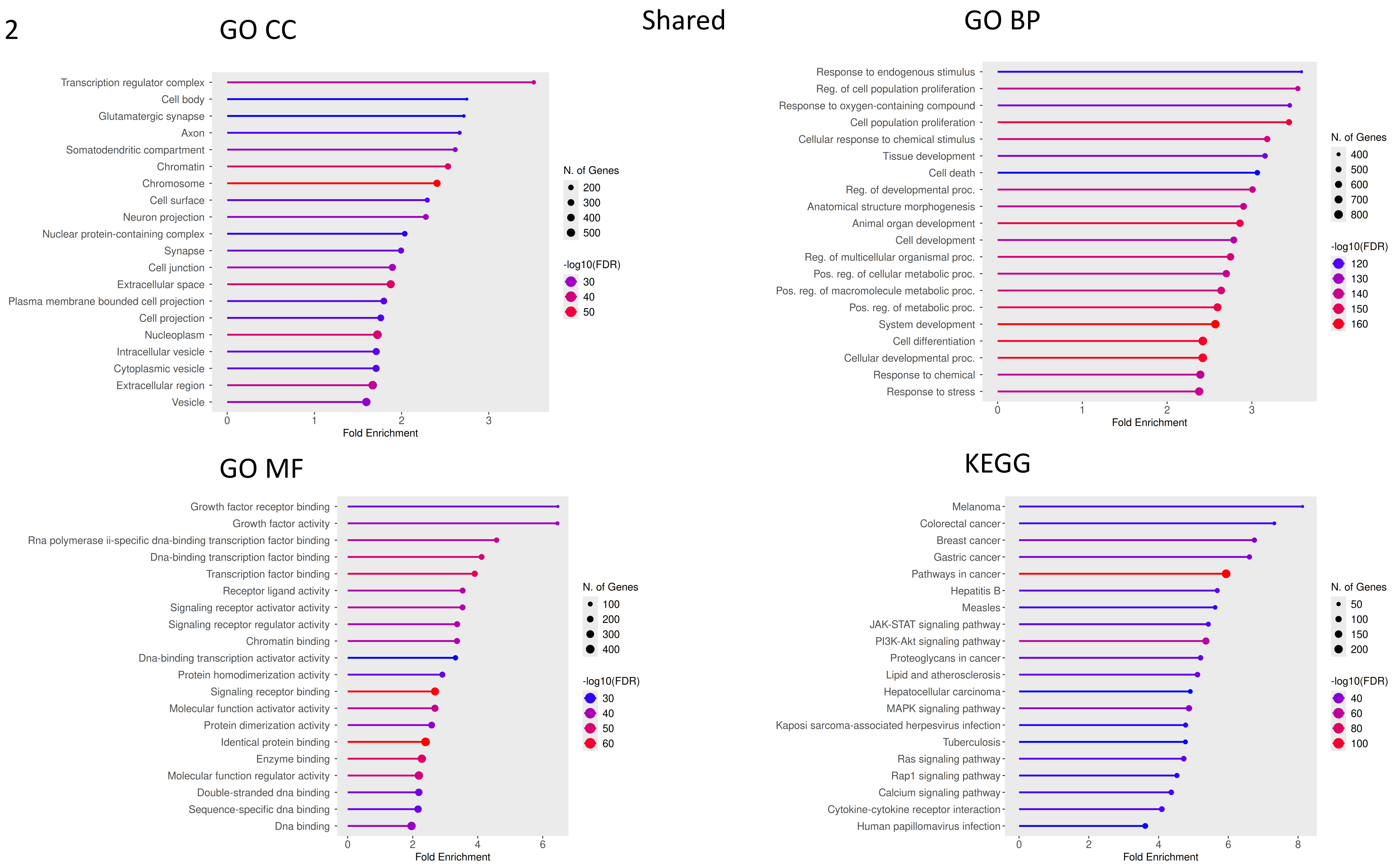
2
Shared
GO CC
Transcription regulator complex
Cell body
Glutamatergic synapse
Axon
Somatodendritic compartment
Chromatin
Chromosome
Cell surface
Neuron projection
Nuclear protein-containing complex
Synapse
Cell junction
Extracellular space
Plasma membrane bounded cell projection
Cell projection
Nucleoplasm
Intracellular vesicle
Cytoplasmic vesicle
Extracellular region
Vesicle
0
1
2
3
Fold Enrichment
N. of Genes
200
300
400
500
-log10(FDR)
30
40
50
GO BP
Response to endogenous stimulus
Reg. of cell population proliferation
Response to oxygen-containing compound
Cell population proliferation
Cellular response to chemical stimulus
Tissue development
Cell death
Reg. of developmental proc.
Anatomical structure morphogenesis
Animal organ development
Cell development
Reg. of multicellular organismal proc.
Pos. reg. of cellular metabolic proc.
Pos. reg. of macromolecule metabolic proc.
Pos. reg. of metabolic proc.
System development
Cell differentiation
Cellular developmental proc.
Response to chemical
Response to stress
0
1
2
3
Fold Enrichment
N. of Genes
400
500
600
700
800
-log10(FDR)
120
130
140
150
160
GO MF
Growth factor receptor binding
Growth factor activity
Rna polymerase ii-specific dna-binding transcription factor binding
Dna-binding transcription factor binding
Transcription factor binding
Receptor ligand activity
Signaling receptor activator activity
Signaling receptor regulator activity
Chromatin binding
Dna-binding transcription activator activity
Protein homodimerization activity
Signaling receptor binding
Molecular function activator activity
Protein dimerization activity
Identical protein binding
Enzyme binding
Molecular function regulator activity
Double-stranded dna binding
Sequence-specific dna binding
Dna binding
0
2
4
6
Fold Enrichment
N. of Genes
100
200
300
400
-log10(FDR)
30
40
50
60
KEGG
Melanoma
Colorectal cancer
Breast cancer
Gastric cancer
Pathways in cancer
Hepatitis B
Measles
JAK-STAT signaling pathway
PI3K-Akt signaling pathway
Proteoglycans in cancer
Lipid and atherosclerosis
Hepatocellular carcinoma
MAPK signaling pathway
Kaposi sarcoma-associated herpesvirus infection
Tuberculosis
Ras signaling pathway
Rap1 signaling pathway
Calcium signaling pathway
Cytokine-cytokine receptor interaction
Human papillomavirus infection
0
2
4
6
8
Fold Enrichment
N. of Genes
50
100
150
200
-log10(FDR)
40
60
80
100

3

EXTRA PULMONAR

GO CC

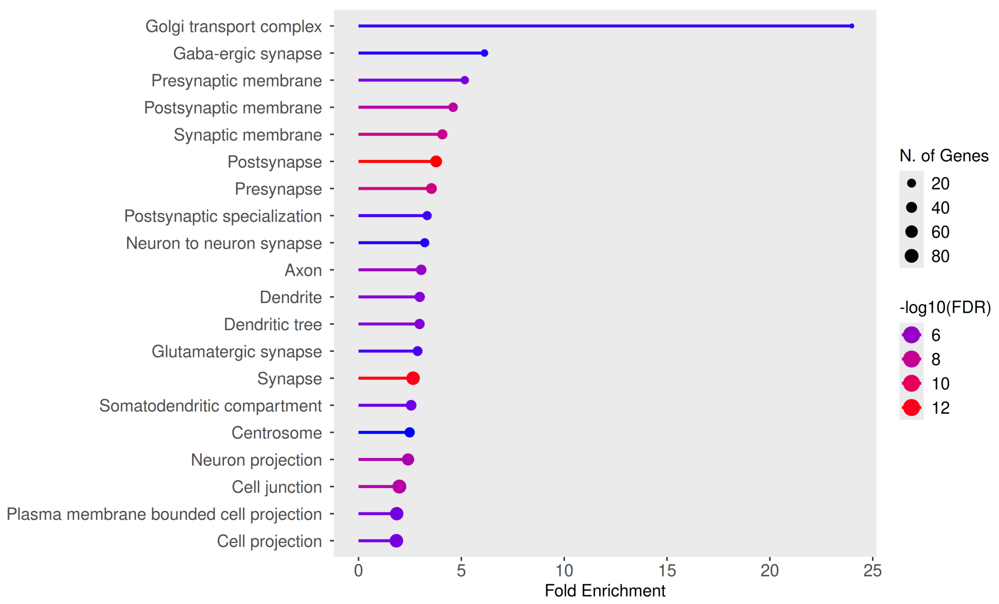

GO BP

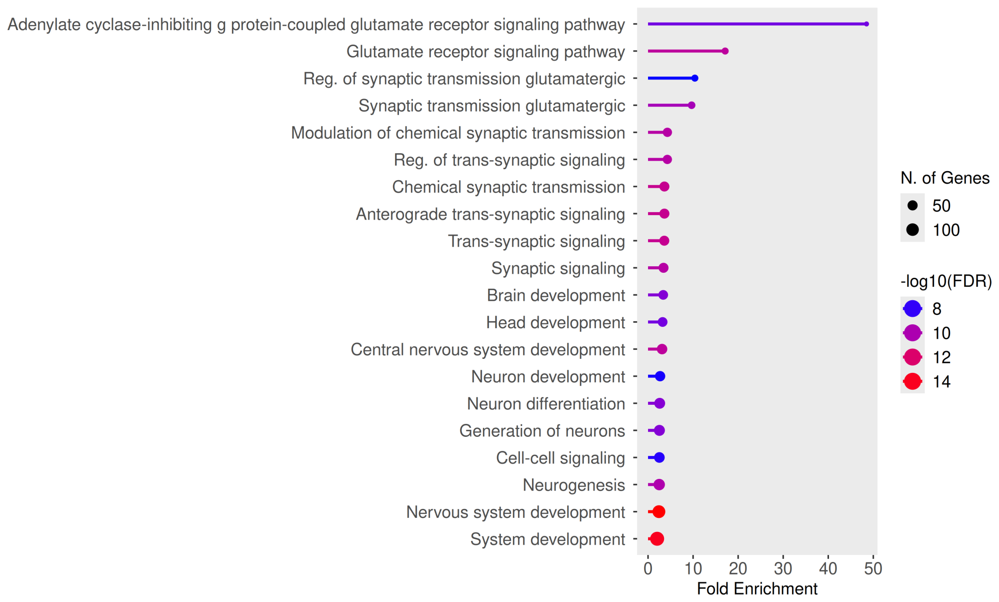

GO MF

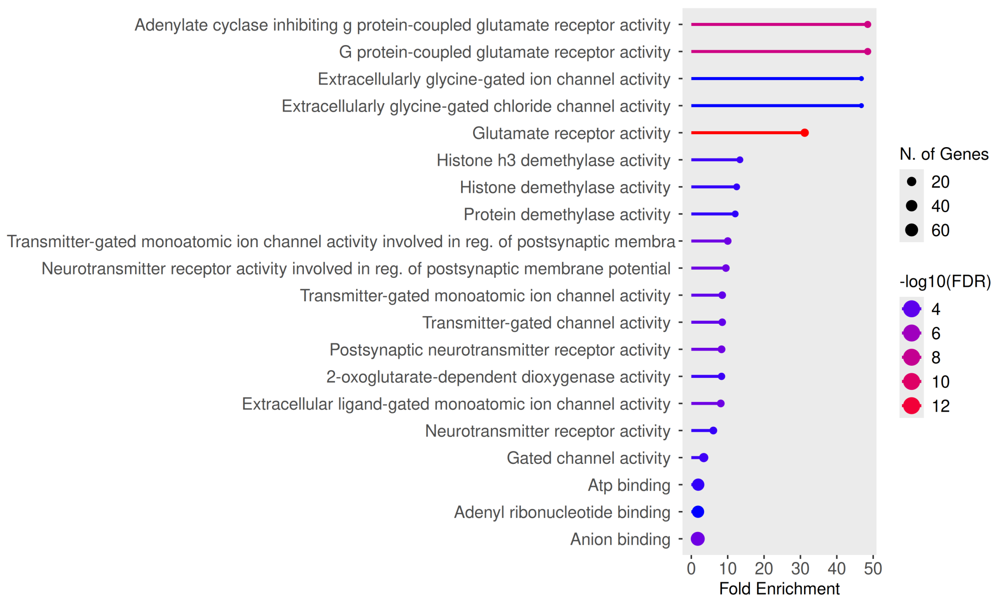

KEGG

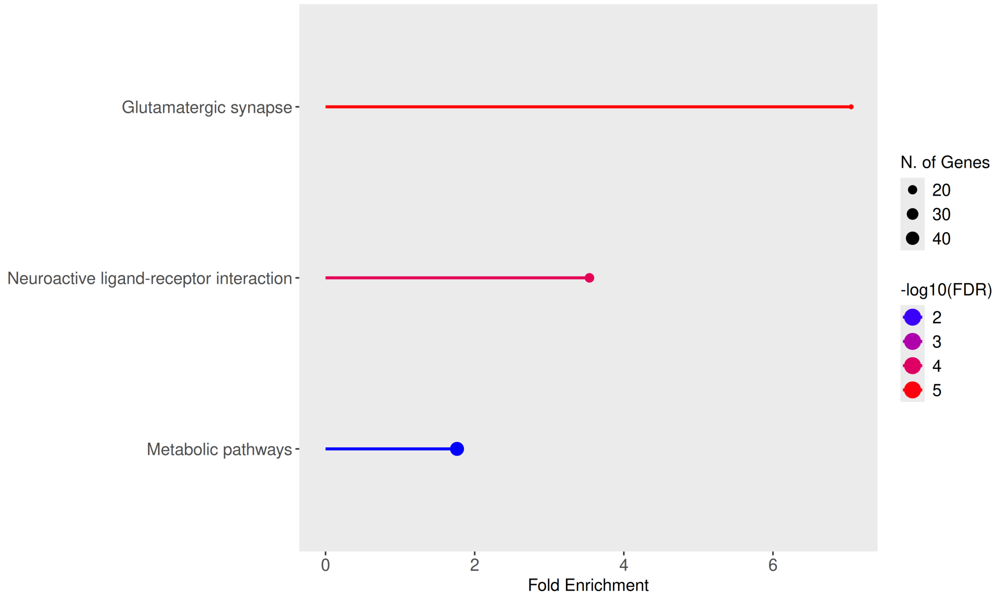

Supplement: Supplementary file 1 [file biology-15-01044-s001.zip › Supplementary Figures.pdf]
